# A Live 13 Weeks Ruptured Ectopic Pregnancy: A Case Report

**DOI:** 10.7759/cureus.10993

**Published:** 2020-10-16

**Authors:** Rawan Gari, Reham Abdulgader, Ossamah Abdulqader

**Affiliations:** 1 Obstetrics and Gynecology, Faculty of Medicine, King Abdulaziz University Hospital, Jeddah, SAU; 2 Obstetrics and Gynecology, King Abdulaziz University Hospital, Jeddah, SAU

**Keywords:** ectopic pregnancy, ruptured ectopic pregnancy, tubal pregnancy

## Abstract

Ectopic pregnancy is a pregnancy that occurs outside the uterus, most commonly in the fallopian tube. It is usually suspected if a pregnant woman experiences any of these symptoms during the first trimester: vaginal bleeding, lower abdominal pain, and amenorrhea. An elevated BhCG level above the discriminatory zone (2000 mIU/ml) with an empty uterus on a transvaginal ultrasound is essential for confirming ectopic pregnancy diagnosis. Such pregnancy can be managed medically with methotrexate or surgically via laparoscopy or laparotomy depending on the hemodynamic stability of the patient and the size of the ectopic mass. In this case study, we report on a 38-year-old woman, G3P2+0 who presented to King Abdulaziz University Hospital’s emergency department with a history of amenorrhea for three months. She was unsure of her last menstrual period and her main complaint was generalized abdominal pain. Upon examination, she was clinically unstable and her abdomen was tender on palpation and diffusely distended. Her BhCG level measured 113000 IU/ml and a bedside pelvic ultrasound showed an empty uterine cavity, as well as a live 13 weeks fetus (measured by CRL). The fetus was seen floating in the abdominal cavity and surrounded by a moderate amount of free fluid, suggestive of ruptured tubal ectopic pregnancy. The patient’s final diagnosis was live ruptured 13 weeks tubal ectopic pregnancy which was managed successfully through an emergency laparotomy with a salpingectomy.

## Introduction

Ectopic pregnancy is a pregnancy in which the developing blastocyst implants outside the endometrial cavity [1]. Extrauterine pregnancy is estimated to account for 1.3% to 2.4% of all pregnancies [2]. 90% of ectopic pregnancies occur in the fallopian tubes, and the remaining implant on the cervix, the ovary, the myometrium, and other sites [3]. Ectopic pregnancy may present as abdominal or pelvic pain, amenorrhea with or without vaginal bleeding in the first trimester. The minimum diagnostic requirement for an ectopic pregnancy is a transvaginal ultrasound and serological confirmation of pregnancy [4]. This article involves an unusual case of a live ruptured 13 weeks ectopic pregnancy which was seen, diagnosed, and managed at King Abdulaziz University Hospital in Jeddah, Saudi Arabia.

## Case presentation

A 38-year-old Filipino patient, G3P2+0 presented to the emergency department on the 18th of October 2019 complaining of acute onset of lower abdominal pain associated with a history of amenorrhea for three months. She was unsure of the date of her last menstrual period and had no previous antenatal follow-up. She was medically free and her past obstetric history included a normal uncomplicated vaginal delivery, followed by a cesarean section which was performed four years back. She had no allergies and was not taking any medication or contraception. Upon presentation, she complained of generalized lower abdominal pain which was of a sudden onset, continuous, not radiating, and not relieved by oral analgesia. The pain was associated with nausea and symptoms of anemia such as dizziness and shortness of breath, but there was no history of loss of consciousness, gastrointestinal or urinary tract symptoms. There was no history of fever or symptoms suggestive of pelvic inflammatory disease. 

**Figure 1:**
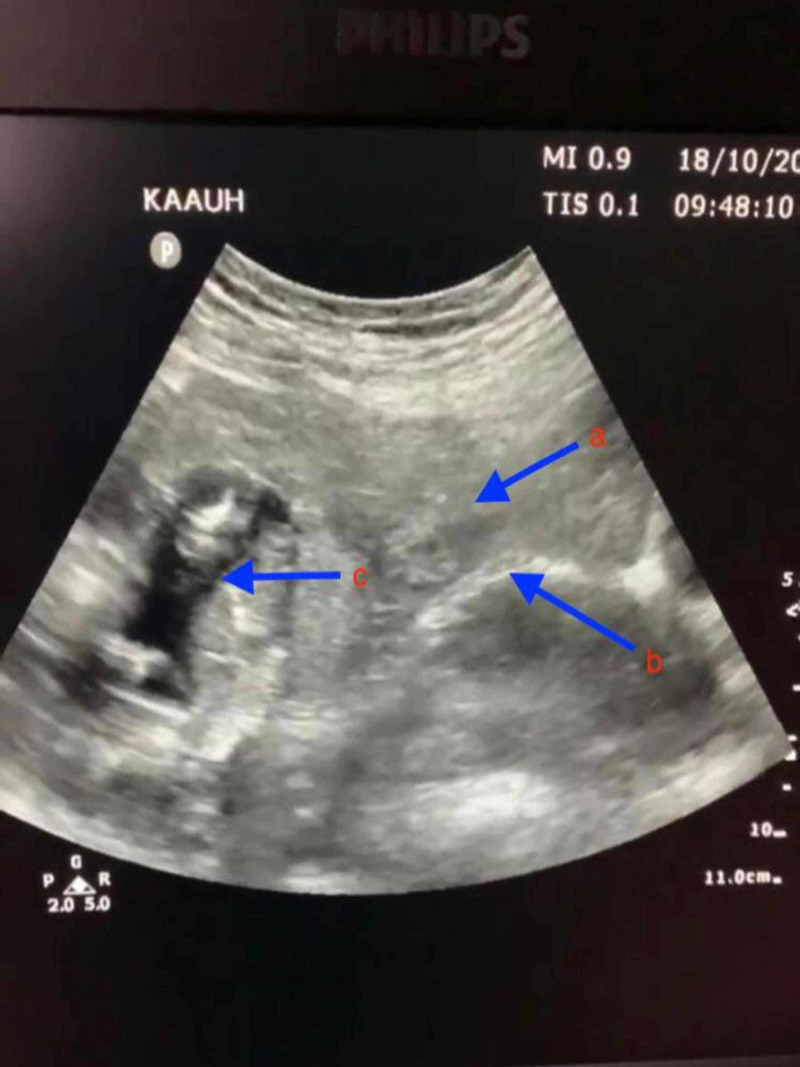
Trans-abdominal ultrasound of pelvis Arrows indicate: (a) uterus; (b) endometrial line; (c) fetus.

Upon clinical examination, the patient looked pale and distressed. Her blood pressure was 90/42 mmHg, with a pulse rate of 110 beats per minute. Her abdomen was generally distended and tender on both superficial and deep palpation, with signs suggestive of peritonitis. The digital vaginal examination was positive for cervical motion tenderness and her BhCG Level measured 113000 IU/ml. The examination was complemented by a bedside pelvic ultrasound, which showed an empty uterine cavity as well as a live fetus floating in a moderate amount of free fluid in the pouch of Douglas (Figure 1). Her hemoglobin count measured 3.2 g/L, and her total white cell count was 7.5 g/L. Blood grouping and cross-matching of four blood units were immediately sent. 

**Figure 2:**
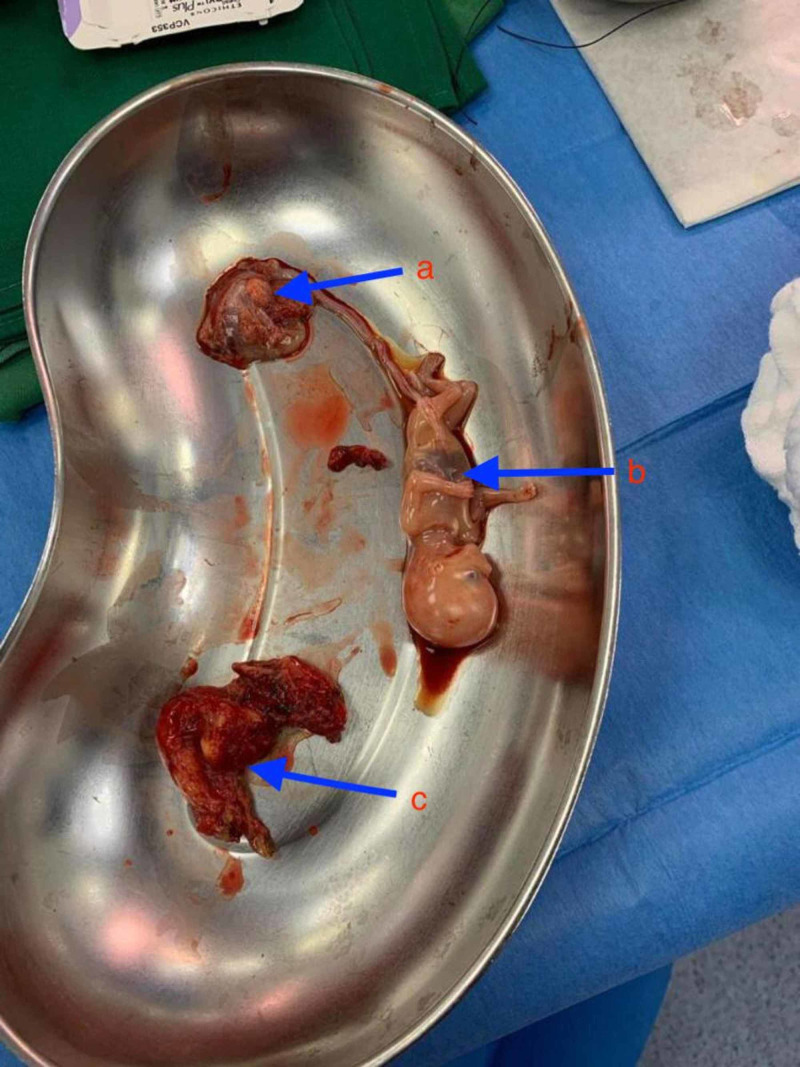
Intra-operative finding Arrows indicate: (a) placenta; (b) fetus; (c) right fallopian tube.

The possibility of a ruptured ectopic pregnancy was explained to the patient, and she consented to an emergency laparotomy with possible salpingectomy. During the laparotomy, a total of 4 liters of intra-abdominal blood was suctioned while blood transfusion was ongoing. A live 13-week fetus was found and removed from the pelvic cavity, and the remains of the ectopic pregnancy (gestational sac and placenta) were found along a ruptured right fallopian tube. The right tube was successfully resected, and the specimen was sent to histopathology. Both the right and left ovaries looked normal. Peritoneal lavage was completed, and a large pelvic drain was inserted. The histopathology report revealed chorionic villi within the lumen of the right tube, which was consistent with tubal ectopic pregnancy (Figure 2).

Intra-operatively, the patient received a total of five units of packed red blood cells plus three units of fresh frozen plasma. She was transferred to the Surgical Intensive Care Unit where she was observed for two days. During her ICU stay, she remained hemodynamically stable. Her oxygen saturation was maintained with a 6L O2 face mask. Her chest was clear with bilateral equal air entry. Her abdomen was soft and lax, and the surgical wound was covered with a dressing. The pelvic drain contained hemoserous fluid measuring around 450cc and urine output was adequate. Repeated hemoglobin level post-transfusion was 10 g/L, and her white blood cell count was 15 g/L. Electrolytes were balanced and double antibiotic coverage was initiated along with and anti-stress medications. On post-op day 3, the patient was transferred back to the Gyne ward. She was discharged home in a stable condition five days after surgery.

## Discussion

Ectopic pregnancy is a well-known first-trimester pregnancy complication. It is a potentially life-threatening condition and is still regarded as a major cause of maternal mortality, as it is responsible for 9% to 13% of all pregnancy-related deaths [2].

The vast majority of ectopic pregnancies implant at different locations in the fallopian tube, most commonly in the ampulla (70%), followed by the isthmus (12%), fimbria (11.1%), and interstitium (2.4%) [5].

Many risk factors are correlated with ectopic pregnancy such as previous ectopic pregnancy, tubal damage or adhesions from pelvic infection or prior abdomino-pelvic surgery, history of infertility, in vitro fertilization treatment, increased maternal age and smoking. However, half of the women with ectopic pregnancies have no identifiable risk factors [6].

Tubal pregnancy often becomes symptomatic in the first trimester due to the lack of submucosal layer within the fallopian tube wall which enables ovum implantation within the muscular wall, allowing the rapidly proliferating trophoblasts to erode the muscularis layer. This usually causes tubal rapture at 7.2 weeks ± 2.2, leading to hemorrhage and shock. However, cases of advanced gestational age with different presentations have been reported in the literature. Such events are rare as it is unusual for the fallopian tube to dilate to the point of accommodating a second- or third-trimester fetus [5].

Ectopic pregnancy remains a challenging diagnosis in an emergency department setting. Therefore, biochemical investigation (BhCG) and skilled sonographic evaluation of the pelvis in a patient with a suspected ectopic pregnancy play a vital role in accelerating the management of patients [7].

Deciding on the best treatment option depends on various factors including the patient’s hemodynamic stability, BhCG level, the size of the gestational sac, and patients’ desire for future fertility. Un-ruptured single ectopic pregnancies can be successfully treated with systemic methotrexate [2]. In our case, an emergency laparotomy and a right salpingectomy were performed due to the ruptured ectopic mass, unstable hemodynamic status of the patient, and the accumulation of a large amount of intra-abdominal blood noted on the ultrasound image.

## Conclusions

Although it is unusual for an ectopic pregnancy to persist beyond the first trimester, it can occasionally occur. Thus, in all cases of surgical abdominal emergencies during pregnancy, it is paramount to rule out ruptured ectopic pregnancy as it is life-threatening to the mother when the proper diagnosis and management are delayed.

## References

[1] (2018). American College of Obstetricians and Gynecologists ACOG practice bulletin no. 191. Tubal ectopic pregnancy. Obstet Gynecol.

[2] Taran FA, Kagan KO, Hübner M, Hoopmann M, Wallwiener D, Brucker S (2015). The diagnosis and treatment of ectopic pregnancy. Dtsch Arztebl Int.

[3] Panelli DM, Phillips CH, Brady PC (2015). Incidence, diagnosis and management of tubal and nontubal ectopic pregnancies: a review. Fertil Res Pract.

[4] Belics Z, Gérecz B, Csákány MG (2014). Early diagnosis of ectopic pregnancy. Orv Hetil.

[5] Khalil MM, Shazly SM, Badran EY (2012). An advanced second trimester tubal pregnancy: case report. Middle East Fertil Soc J.

[6] Diarra M, Guèye N, Guèye M, Thiam I, Mbaye M, Magib A (2013). Unruptured tubal pregnancy in the second trimester. South Sudan Med J.

[7] Santos L, Oliveira S, Rocha L (2020). Interstitial pregnancy: case report of atypical ectopic pregnancy. Cureus.

